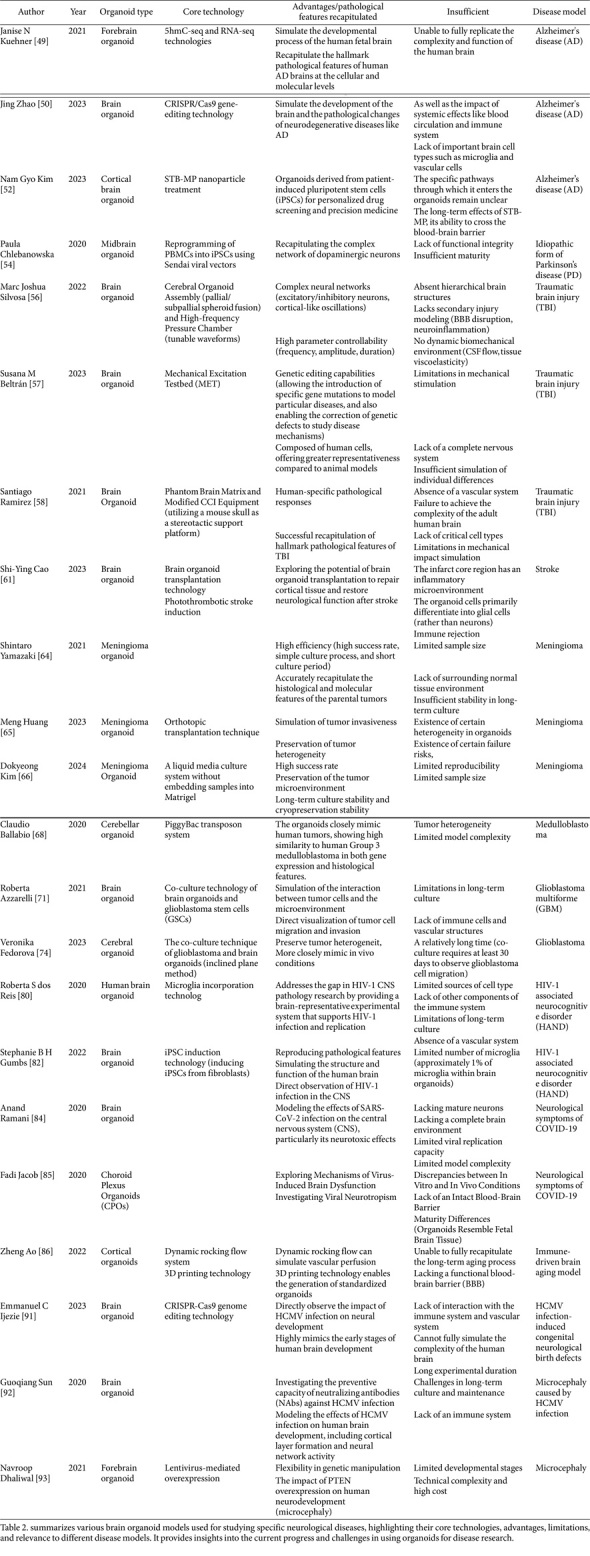# Human Brain Organoids: Development and Applications

**DOI:** 10.4014/jmb.2411.11040

**Published:** 2025-05-28

**Authors:** Wu Hongxi, Wang Ruting, Liu Yiyang, Huang Qinglian, Chen Jibing, Jiang Feng

**Affiliations:** 1 Graduate School, Guangxi University of Chinese Medicine, Nanning, Guangxi, P.R. China; 2 Ruikang Hospital, Guangxi University of Chinese Medicine, Nanning, Guangxi, P.R. China; 3 Affiliated Hospital, Guangdong Medical University, Zhanjiang, Guangdong, P.R. China

**Keywords:** Brain organoids, human pluripotent stem cells, disease modeling, neurological disease, brain development

## Abstract

Brain organoids are three-dimensional structures generated from pluripotent stem cells, closely resembling the embryonic human brain. They exhibit gene expression patterns and signaling pathways similar to those in the developing human brain, facilitating the study of the molecular mechanisms underlying brain development, as well as genetic and environmental factors underlying developmental disorders. A brain organoid comprises various cell types noted in a developing brain: neurons, astrocytes, microglia, and oligodendrocytes. These cells interact with each other and form complex networks, enabling the investigation of communication among different cell types and their contribution to brain function. Brain organoid structure is also similar to that of a developing human brain, with distinct features resembling regions such as the cortex and ventricles. Alternatively, organoid models cannot completely replicate certain specific stages of brain development, such as brain surface folding and complex neuronal circuitry establishment. Nevertheless, few advancements to improve organoid systems and mimic embryonic human brain complexities have been reported. These include sophisticated culture protocol establishment, brain organoid vascularization and transplantation, regionalized brain organoid assembly into relatively complete brain organoids (*e.g.*, assembloids). In general, recent advancements in brain organoid technology have demonstrated significant potential for advancing regenerative medicine, drug discovery, and disease modeling.

## Introduction

Human brain formation is a complex process influenced by genetic and environmental factors. Moreover, the completion of human brain development, including complex connection establishment and cognitive and behavioral ability acquisition, requires many years [1]. Genetic factors play a major role in brain development and are closely associated with various developmental programs in the human body [2]. However, the current understanding of the molecular and circuit mechanisms underlying neuropsychiatric disorders due to genetic factors remains lacking. Therefore, alternative experimental systems replicating the human context are warranted. The development of protocols for human stem cell culture and differentiation has revolutionized the in vitro study of human cells. This technology allows researchers to study human development and diseases accurately, explore potential treatment options, and investigate fundamental biological processes in the human system-all without the limitations and ethical concerns associated with animal models. This progress in stem cell technology holds great promises for regenerative medicine, drug discovery, and disease modeling, as well as for advancing our understanding of human biology.

The absence of tissue architecture and environment in traditional culture systems, nevertheless, limits the accurate study of the disease phenotypes. Typical animal cell cultures involve growing cells on flat surfaces, lacking the intricate three-dimensional (3D) structures found in actual tissues. As such, researchers have developed suspension culture techniques using mycoplasma and virus-free induced pluripotent stem cell (iPSC) lines without common genetic duplications or deletions. Based on iPSCs’ ability to self-organize and differentiate, they have developed innovative culture protocols for 3D brain organoids [3]. These techniques can effectively create complex structures similar to those found in a developing human brain, such as those of the pituitary gland [4, 5], striatum [6], retina [7, 8], and cortex [9].

In this review, we introduce and discuss recent improvements in brain organoid models and technologies and their applications as model systems and discovery tools, as well as explore the relevant challenges and limitations. In general, we provide a balanced view of the advantages and limitations of brain organoids.

This review systematically examines brain organoid research through a literature search (2018–present) in PubMed/Web of Science using standardized terms including "brain organoid", "cerebral organoid", "disease model*", and others (full strategy: Table S1). We analyze technological advancements in organoid modeling, their dual roles in disease recapitulation and mechanistic discovery, and persistent limitations, with comparative evaluations provided in Table 1 (methodological benchmarks) and Table 2 (disease-specific applications). The term 'brain organoid' is used throughout this review to align with prevailing terminology in the field, though some studies referenced may use 'cerebral organoid' in their original descriptions.

## Brain Organoid Cultivation Method Development

**Fig. 1:**
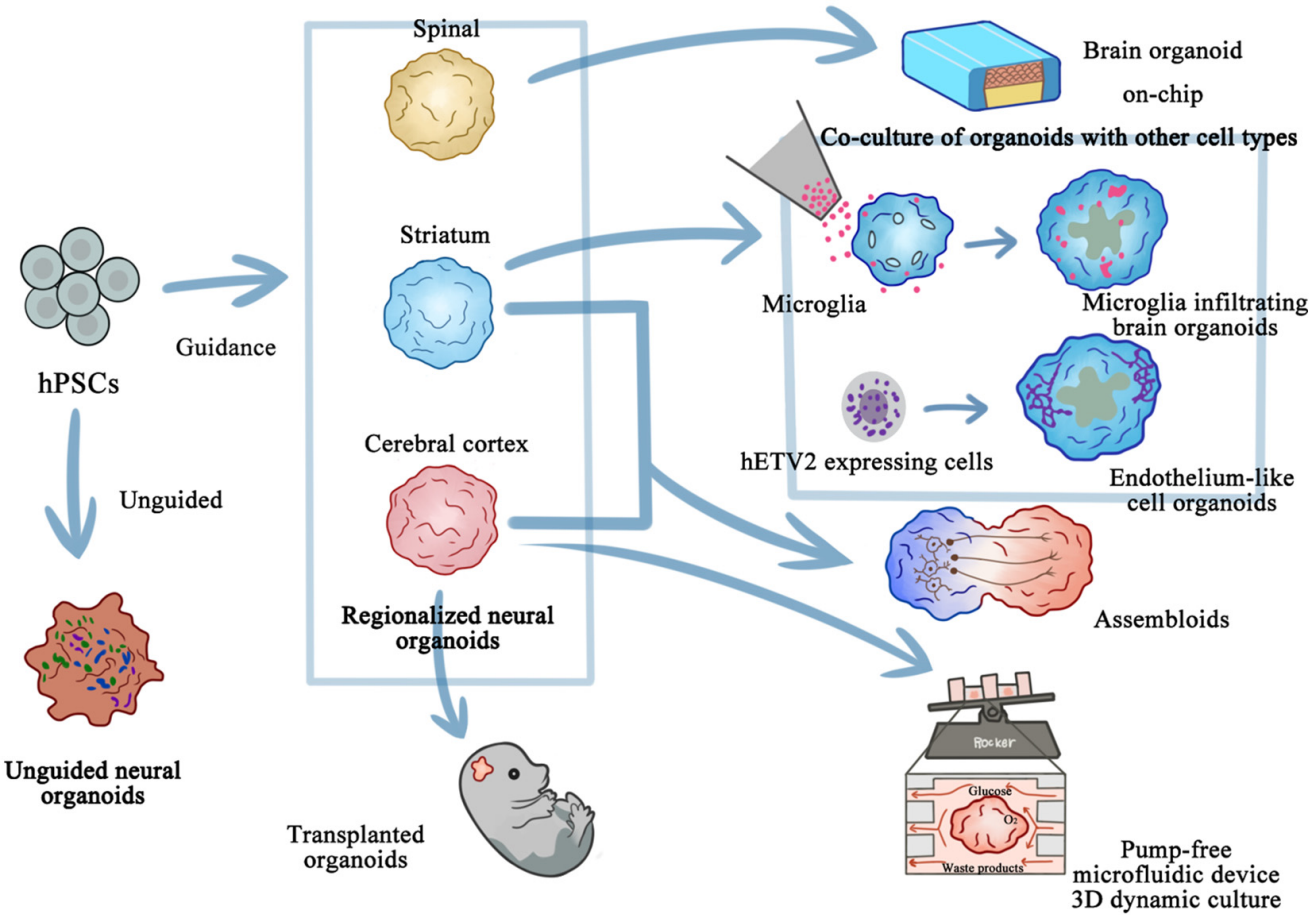
Schematic workflow for cerebral organoid generation, diversification, and advanced co-culture applications (The figure is adapted from reference [11]). This illustration outlines a comprehensive pipeline for deriving region-specific brain organoids from human pluripotent stem cells (hPSCs) via guided (*e.g.*, spinal cord, striatum, cerebral cortex) or unguided neural differentiation, followed by their functional integration in disease modeling and bioengineering systems. Left panel: hPSCs differentiate into neural organoids with regional identities (spinal, cortical, or striatal) through morphogen-guided patterning or self-organization (unguided). Central panel: Organoids are co-cultured with microglia (infiltrating immune cells), hETV2-expressing endothelial progenitors, or endothelium-like cell organoids to model neuroimmune interactions or vascularization. Right panel: Advanced applications include assembloid fusion (*e.g.*, neural-vascular assembloids), transplantation into host tissue, and pump-free microfluidic 3D dynamic culture systems (featuring a rocker-driven, waste-efficient chip) for long-term maturation and perfusion studies. Color-coded annotations distinguish cell types (microglia, endothelial cells), differentiation pathways (guided, unguided), and bioreactor components (microfluidic chip). The figure indicate dynamic cross-system interactions, emphasizing scalability for neurodegenerative disease modeling and blood-brain barrier research.

Lancaster *et al*. [10] reported a modified approach to generate neuroectoderm from embryoid bodies. The authors cultivated neuroectodermal tissues in a 3D culture and embedded them in Matrigel droplets, which served as scaffolds for complex tissue growth. To enhance nutrient absorption, these Matrigel droplets were transferred to a spinning bioreactor. This resulted in the rapid development of brain tissues, which the authors referred to as brain organoids. Furthermore, Pașca *et al*. [11] proposed the use of the terms “regionalized neural organoids” and “unguided neural organoids” to describe self-organizing neural organoids, depending on the level of guidance used during the pluripotent stem cell differentiation process. When these organoids are combined with other organoids or specialized cell types, they form assembloids. Furthermore, when transplanted into animals, these assembloids are called grafted organoids or assembloids (or transplanted organoids or assembloids). Assembloids may also be created by combining organoids from different individuals (interindividual assembloids) or even different species (interspecies assembloids). Effective and precise for brain organoid cultivation methods other than those mentioned above have been reported. These include employing engineering approaches such as rotating cell-culture systems and microfluidic platforms, all of which improve organoid quality and survival rate [12]. Moreover, researchers have developed engineered extracellular matrices [13] and multiplex platforms [9] to enhance cell-matrix interactions and create controlled environments. Furthermore, detailed protocols have been developed to generate organoids with specific brain identities. The specific procedures and methodologies for the cultivation of brain organoids are delineated in Fig. 1.

## Regionalized Neural Organoids Versus Unguided Neural Organoids

Neural organoids can be categorized into two main groups based on the level of guidance provided during the differentiation of pluripotent stem cells into organoids. Here, the term “guidance” refers to the addition of small molecules or factors intended to induce the formation of specific cell-type regions or collections. The term “guided” is used to describe this process, as exogenous factors are employed to guide the self-organization and self-patterning in 3D cultures toward development into a particular region of the nervous system. Unguided organoid differentiation results in organoids with high neural cell diversity, representing various neural axis locations and nonneural derivatives; this method, therefore, results in unguided neural organoids [11]. In contrast, regionalized neural organoids, composed of more regionally specific cell types, are constructed using instructive signals [14, 15]. These organoids can mimic the cellular, molecular, or anatomical features of particular nervous system domains. For instance, hypothalamic, pituitary, striatum, sensorimotor, cerebellar, and cerebral cortical organoids resemble parts of the hypothalamus [4, 11, 16], pituitary gland [4], striatum [6], neuromuscular junctions [17], cerebellum [18-20], and dorsal forebrain [21-23], respectively. Eigenhuis *et al*. [24] developed a detailed protocol for generating brain organoids with cortical identity from iPSCs. Their self-patterning approach minimizes media supplement use and handling steps, resulting in cortical brain organoids that can be maintained over extended periods. These organoids contain various types of neurons and astrocytes, providing a more accurate representation of the cortical brain structure.

## Assembloids

An assembloid is composed of an organoid combined with other organoids or specialized cell types in a 3D culture [11, 25]. Assembloids enable the modeling of various processes such as neuroimmune interactions, cell migration, and the assembly of neural circuits. They can be generated by incorporating different organoids into multiregion assembloids or by integrating neural or nonneural cells into the organoids. They can also be created from organoids derived from different individuals or species, resulting in interindividual or interspecies assembloids. Mosaic organoids comprise cells from different individuals or a combination of mutant and wildtype cell lines. Assembloids have been used to effectively model complex interactions among different brain regions and study neurodevelopmental defects. A blood–brain barrier (BBB) organoid cocultured with a brain organoid can provide insight into the BBB’s role in brain development [26]. Researchers have also developed methods to induce hypothalamic and pituitary tissues from human pluripotent stem cells (hPSCs) and studied specific brain regions [4]. Using a 3D coculture system with meningeal cells leads to better organization of cortical brain structures for the study of neurodevelopmental disorders. Microfluidic technology can also be used for the codevelopment of cells, leading to the formation of organized vascular networks that interact with brain organoids [27]. Moreover, brain region–specific organoids can be assembled into assembloids by using a multilayered microfluidic chip, enabling neural migration and interaction modeling [28]. Assembloids have also been used to model pancreatic cancer (PC) nerve invasion and study potential therapeutic strategies [29]. This progress in assembloid research may revolutionize the current understanding of brain development, function, and neurological diseases.

## Transplanted Organoids

Transplanted (or grafted) organoids can integrate within an animal’s system better than other methods when human cells are incorporated into early embryonic stages [11]. A study using newborn athymic rats found that transplanted organoids can develop into mature cell types and integrate into sensory and motivation-related circuits [30]. Another study showed that human forebrain organoids transplanted into rats’ visual systems could restore visual function [31]. Reciprocally, a limitation related to these approaches was the lack of modeling interactions between the human brain environment and microglia. As such, researchers have developed an in vivo xenotransplantation approach to study functionally mature human microglia within a human brain organoid model, facilitating further understanding of their role [32]. Moreover, transplanted human cortical organoids in rats have been shown to integrate into sensory and motivation-related circuits, allowing for the study of cell interactions and contributions to brain functioning [33]. In addition, Ibanez-Rios *et al*. introduced a cost-effective, readily available model for transplanting and monitoring human neural cells in human brain organ tissues, providing a straightforward and affordable solution for long-term tracking of transplanted cells [34]. Furthermore, Jgamadze *et al*. proposed a protocol for transplanting forebrain organoids into the optic cortex of injured adult rats to investigate the efficacy of organoids as structural grafts for neural repair [35]. In summary, research has demonstrated that transplanted organoids have great applicability in regenerative medicine; it is expected to revolutionize the conventional approaches of organ transplantation and promote development and innovation in regenerative medicine. In the near future, these results may provide patients with more reliable, safe, and effective treatment options.

## Brain Organoid Vascularization

**Fig. 2:**
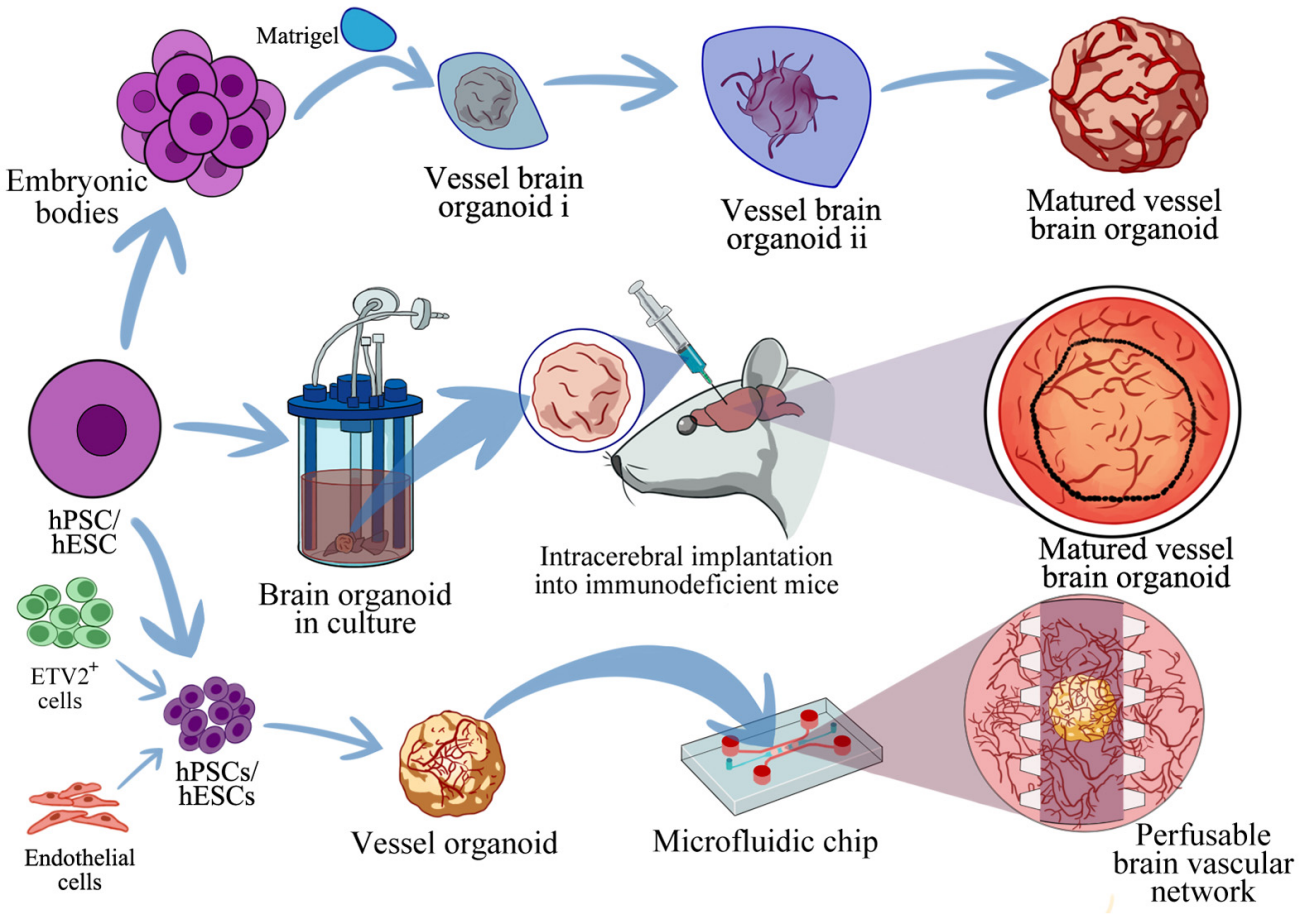
Diverse strategies for vascularizing cerebral organoids. (The figure is adapted from references [37-38, 41]). 1. Conventional in vitro vascularization: hPSCs/hESCs are aggregated into embryoid bodies and embedded in Matrigel to promote neural and vascular co-differentiation, progressing from primitive vascularized organoids (vessel brain organoid i and vessel brain organoid ii) to matured vessel-enriched cerebral organoids through sequential hypoxia-driven endothelial maturation. 2. In vivo transplantation: Pre-vascularized brain organoids are intracerebrally implanted into immunodeficient mice, where host-derived endothelial cells infiltrate and remodel the organoid vasculature, resulting in functional perfusion and complex vascular networks integrated with the host circulatory system. 3. Microfluidic-engineered perfusion: ETV2+-induced endothelial cells or primary endothelial cells are co-cultured with hPSC/hESC-derived neural progenitors to form vessel organoids, which are integrated into a microfluidic chip. Under controlled flow dynamics, endothelial cells migrate, anastomose, and form perfusable 3D vascular networks that mimic the brain’s neurovascular architecture. Key components: Matrigel (structural scaffold), embryoid bodies (pluripotent cell aggregates), vessel organoids (ETV2+ or endothelial cell-derived), and microfluidic chips (vascular patterning tools).

One of the human brain organoid vascularization approaches involves the transplantation of the organoids into immunodeficient rats [36, 37]. Alternatively, replicating this procedure on a larger scale has been challenging because of the inherent molecular and genetic differences between human and rat cells. Another vascularization approach involves the incorporation of vasculature-deriving cells into the brain organoids. This can be achieved by coculturing human umbilical vein endothelial cells (HUVECs) with hiPSCs or human embryonic stem cells (hESCs) [38]. With this process, vascular system formation can be effectively induced within brain organoids, resulting in the development of brain organoids with HUVEC-derived functional blood vessels. Manipulating hESCs to overexpress a specific protein, human ETS variant 2, leads to the formation of vascularized brain organoids with a complex network resembling a human brain’s vasculature [39]. These vascularized brain organoids also develop a BBB-like phenotype, and when transplanted into animal models, they can integrate with the host circulatory system. Researchers have also developed vascularized human cortical organoids (vOrganoids), created using various human cortical cell types [38]. vOrganoids remain functional and vascularized for an extended period, accurately mimicking the blood vessel formation process in the human brain. When transplanted into the mouse cortex, these organoids develop functional human–mouse blood vessels within the grafts, enhancing transplanted cell survival. Another study focused on vascularized brain organoid creation through a combination of brain organoids with vascular spheroids [40]. This approach results in Wnt/β-catenin pathway up-regulation, promoting organoid vascularization. To address issues with the current brain organoid cultivation protocols, researchers have begun incorporating bioengineering techniques such as genetic engineering, coculture, multidifferentiation, microfluidics technology, extracellular matrix protein and synthetic material use, and 3D bioprinting [27, 41-44]. These techniques are aimed at promoting brain organoid maturation and creating additional functional models. In particular, microfluidics enables vascular perfusion and curated BBB microenvironment creation, enhancing the vascularization of brain organoids and enabling substance delivery into them [45]. These developments in culturing techniques and methodologies have contributed to the development of relatively reliable in vitro models for studying human central nervous system (CNS) development, disease progression, and translational applications. The techniques and methodologies for the vascularization of brain organoids are extensively detailed in Fig. 2.

## Disease Modeling

**Fig. 3:**
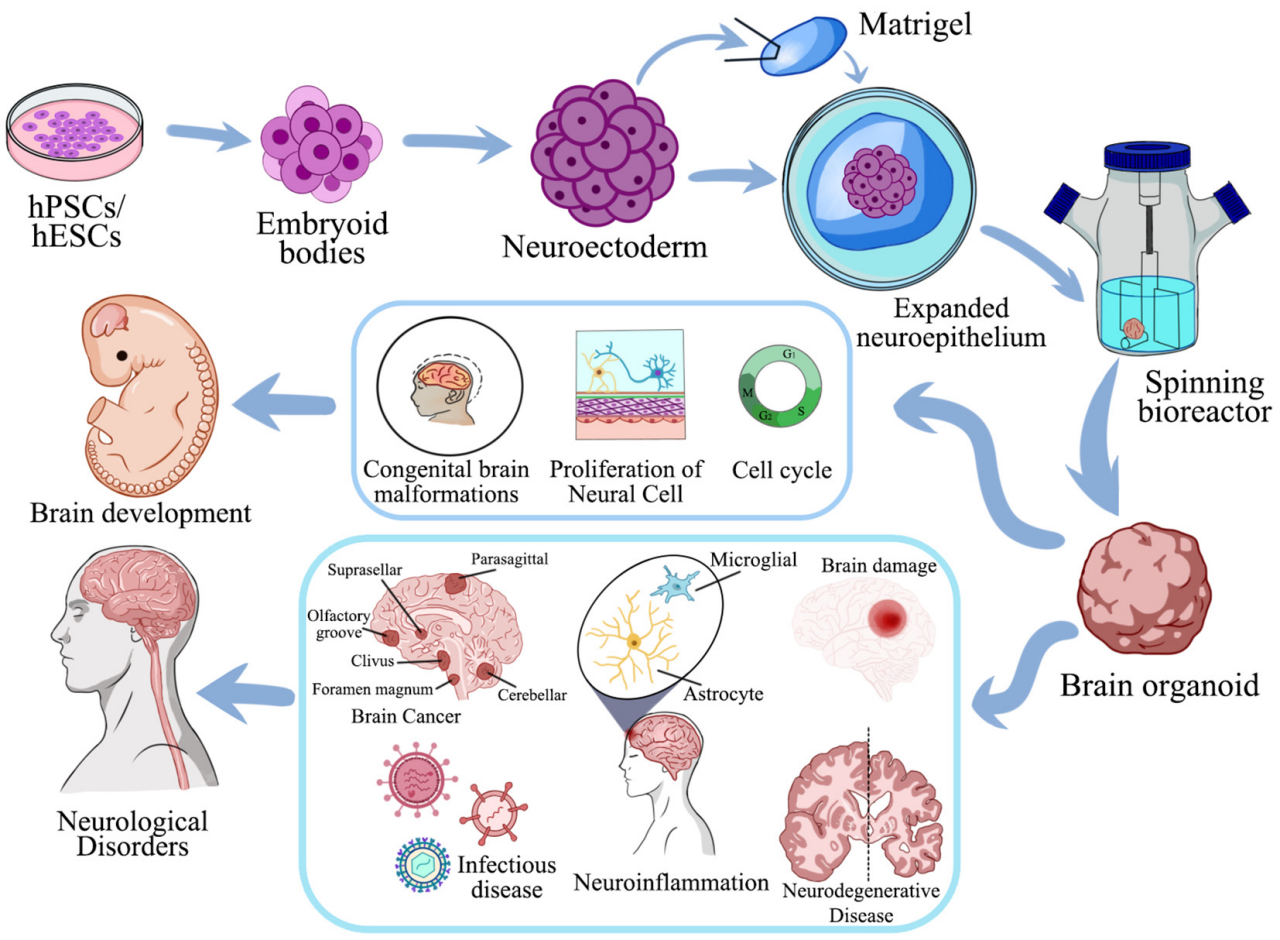
Schematic overview of cerebral organoid derivation from human pluripotent stem cells (hPSCs/hESCs) and their applications in neurological research (The figure is adapted from reference [46]). (Top) Protocol for cerebral organoid generation: hPSCs/hESCs aggregate into embryoid bodies, followed by neural induction to form neuroectoderm. These structures are embedded in Matrigel to promote neuroepithelial expansion and subsequently cultured in a spinning bioreactor to generate 3D cerebral organoids. Key stages include proliferation, cell cycle regulation, and regional specification (*e.g.*, cerebellar, olfactory, and suprasellar regions). (Bottom) Applications in disease modeling: Cerebral organoids recapitulate brain development and enable studies on congenital brain malformations (*e.g.*, clivus or foramen magnum defects), brain cancer, infectious diseases, neuroinflammation (highlighting microglial activation), and neurodegenerative disorders. Astrocyte networks and microglial responses to brain damage are illustrated, emphasizing their roles in neural repair and pathology. Color-coded annotations distinguish cellular components, developmental stages, and disease-specific mechanisms.

Brain organoids derived from hPSCs, particularly patient-derived iPSCs, have been extensively studied as a potential tool for modeling neurodevelopmental brain disorders [46]. These organoids can efficiently recreate disease-related characteristics associated with conditions whereby structural abnormalities occur during early embryonic development stages. The mechanisms underlying such conditions are often allocated to disturbances in the regulation of progenitor cells, including premature differentiation, decreased proliferation, and cell-cycle disruptions. In controlled laboratory settings, brain organoids remain the most reliable and accurate means to analyze these disruptions. As illustrated in Fig. 3, brain organoids enable recapitulation of cerebral development and facilitate investigations into a spectrum of neurological conditions – ranging from congenital brain malformations, brain cancers, and infectious diseases, to neuroinflammation and neurodegenerative disorders.

## Neurodegenerative Disorders

Neurodegenerative disorders [*e.g.*, Alzheimer's disease (AD), Parkinson's disease, and amyotrophic lateral sclerosis] are disorders affecting the CNS or peripheral nervous system, resulting in nerve cell damage and death and in turn leading to a progressive loss of neurological function in various body parts [47]. In vitro study of neurodegenerative disorders involves using brain-like organs, which can be prepared in the laboratory by using methods such as culturing nerve or stem cells from animals [48]. By mimicking disease characteristics and processes, researchers can understand the mechanisms by which they occur and test potential treatments further.

## AD

AD, the most common neurodegenerative disorder, manifests as progressive physical function worsening, behavioral impairment, and cognitive decline. In general, AD is characterized by a gradual decline in brain function and the formation of abnormal protein deposits, such as amyloid β plaques and tau tangles, in the brain. A study [49] analyzed changes in the molecule 5-hydroxymethylcytosine (5hmC) during different stages of brain development in organoids derived from patients with AD. The authors noted dysregulation of 5hmC modifications, providing insight into AD’s pathogenesis. Another study highlighted the critical role of the Apolipoprotein E (APOE) gene in maintaining brain function and identified potential therapeutic targets for AD [50]. Karmirian *et al*. [51] developed a protocol for generating brain organoids from patients with AD and analyzing AD pathology. These AD organoids can replicate the pathological characteristics of amyloid β and tau, making them a valuable tool for studying the molecular mechanisms underlying AD and testing the effects of potential treatments. Moreover, Kim N.G. *et al*. [52] used iPSCs from patients with AD to generate cortical brain organoids expressing AD phenotypes, including amyloid β and hyperphosphorylated tau accumulation, both of which are hallmark features of AD. These studies have contributed to further understanding of AD pathology and effective treatment development.

## Parkinson’s Disease

Parkinson’s disease affects the CNS, leading to progressive motor symptoms such as tremors, muscle stiffness, bradykinesia (*e.g.*, slowness of movement), and postural instability [53]. Parkinson's disease has been studied comprehensively through the creation of organoid models derived from not only patients with Parkinson's disease but also healthy individuals [54]. These organoids closely resemble human brain structure and function and demonstrate differences in Parkinson’s disease marker expression between the two groups. By using these organoids, scientists can study Parkinson's disease progression and explore potential treatments. Moreover, a study used iPSCs derived from patients with neurodegenerative disorders (including Parkinson’s disease) and a neural organoid platform to generate brain organoids specifically for Western Pacific Amyotrophic Lateral Sclerosis and Parkinsonism-Dementia Complex (ALS-PDC) [55]. The results indicated that the patient-specific approach indicated ALS-PDC pathogenesis and potential treatment options, providing valuable insights into future therapeutic improvements.

## Nervous System Traumas

Nervous system traumas occur when the body’s nervous tissue is impacted by an outside force, such as a car accident, fall, or drop. This injury can cause various disorders, such as traumatic brain injury (TBI), brain embolism, and disorders related to the nervous system. When the nervous system sustains an injury, the neurons in it are damaged or lose their function, in turn leading to nerve pathway disruption and dysfunction. Conversely, the emergence of brain-like organs has provided avenues for the study of regenerative medicine for neurological injury treatment. Brain-like organs are organs artificially grown in vitro to resemble normal brain structure and function. By using brain-like organs, researchers have investigated the process of neural development and further repair and explored newer treatment approaches for neurological injuries.

## TBI

A TBI refers to the damage sustained by brain tissue as a result of physical forces originating from the external environment. These physical forces can include a wide range of factors such as a direct impact, collision, fall, or penetrating injury. In recent years, many studies have used human brain organoids to simulate and investigate TBI. Silvosa *et al*. [56] employed a benchtop blast simulator to administer precisely calibrated pressure waveforms to cortical organoids, demonstrating sub-lethal threshold impulses that transiently suppress neuronal network activity while preserving cellular viability. Beltrán *et al*. [57] implemented uniaxial compression paradigms to induce graded mechanical strain, subsequently employing transcriptomic profiling to delineate strain-dependent pathway activation. Notably, Ramirez *et al*. [58] engineered a biofidelic injury platform by encapsulating organoids within a murine skull replica containing a tissue-mimetic hydrogel matrix (agarose-gelatin composite mimicking the viscoelastic properties of murine neural parenchyma). Controlled cortical impact delivered through this hybrid system recapitulated hallmark neuropathological features including astroglial activation, neurite degeneration, and caspase-mediated apoptosis. Furthermore, Kim *et al*. [59] proposed a theoretical framework integrating brain organoids with microfluidic organ-on-a-chip systems through patient-specific iPSCs. The paradigm enables mechanobiological modeling of BBB dysfunction and pathological cascades while mapping interorgan signaling networks post-TBI, establishing a biomimetic platform for neurorestorative mechanism investigation and high-throughput drug screening.

## Traumatic Cerebral Infarction

In traumatic cerebral infarction, cerebral blood vessels are blocked due to external causes, typically resulting in brain artery narrowing or obstruction due to external forces, leading to insufficient blood supply to or ischemia in the brain [60]. The causes underlying this cerebral infarction type may include trauma, cranial surgery, or accidents. Stem cell transplantation may, nevertheless, enhance neuroplasticity and improve neuronal defects due to cerebral embolism. In a groundbreaking study [61], stem cell-derived human brain organoids were transplanted into mice with stroke. Several months after transplantation, the organoids demonstrated resilience and survival in the affected area, differentiated into specific neurons, restored the damaged tissue, and integrated into the existing neural circuitry. Individual cell transplantation did not lead to identical regenerative properties. Therefore, brain organoid transplantation may repair and restore brain functionality affected by cerebral embolism.

## Brain Cancer

Brain cancer is a complex and devastating disease; in spite of that, understanding its biology and genetics and finding effective therapeutic approaches may be significantly challenging. Nonetheless, recent progress in organoid research has provided a promising alternative model for the study of brain cancer [20].

## Meningiomas

Meningiomas are the most common primary tumors of the central nervous system (CNS) [62]. According to the WHO, meningiomas are classified into three grades based on histological characteristics: Grades I, II, and III [63]. In a study, Yamazaki and his team [64] established 16 organoid models using tumor tissues from 16 patients, including 10 Grade I meningiomas, 3 Grade II meningiomas, 1 Grade III meningioma, and 2 solitary fibrous tumors (SFTs), with a success rate of 100%. The surgically resected tissues were minced into 1 mm³ pieces in organoid culture medium or serum-free medium. After removing red blood cells and debris, the isolated cells were resuspended in a matrix gel and diluted with an equal volume of medium. Then, the Matrigel was incubated until it solidified and subsequently covered with medium. The medium was refreshed every few days. These organoids were passaged every 2-4 weeks. Through these organoid models, they found that overexpression of the FOXM1 gene increased the proliferation of Grade I meningioma organoids (MNOs). In contrast, knocking down FOXM1 in malignant MNOs inhibited organoid growth. In vitro experiments using MNO models indicated that they found abnormally upregulated FOXM1 may be an effective therapeutic target. Additionally, Huang's research team [65] discovered through targeting SULT1E1+ in MNOs that the synthetic compound SRT1720 could serve as a potential drug for systemic treatment and radiosensitization. Furthermore, Kim and his team [66] used a liquid culture medium system (instead of embedding samples in Matrigel) to obtain a system that is easy to operate, cost-effective, and time-saving. They established MNOs from four meningioma patients with a 100%success rate. Their MNO models overcame the limitations of previous meningioma models and showed superior similarity to the parent tumors. These MNO models provide more options for promoting the translational research of meningioma drugs in the era of precision medicine.

## Medulloblastoma

Medulloblastoma (MB) is a highly aggressive brain cancer, which primarily affects children [67]. Among all cancers, it is responsible for the highest morbidity and mortality rates. By studying brain cancer organoids generated from genetically modified hiPSC-derived brain organoids, researchers have made considerable progress in understanding the underlying mechanisms and potential treatment options for MB [20]. Among all MB subtypes, Group 3 MBs, characterized by MYC overexpression, are the most aggressive. Moreover, in the cerebellar organoids, OTX2/MYC plays a crucial role in Group 3 MB tumorigenesis. Reciprocally, MB progression can be inhibited by using the EZH2 inhibitor tazemetostat [68].

## Glioblastoma

Glioblastoma is another aggressive form of brain cancer. Study of this disease by using in vitro models has long been challenging because of its infiltrative nature [69]. However, the development of 3D cultured human brain organoids has provided a more accurate, scalable representation of glioblastoma’s infiltrative capacity [70]. These organoids can mimic the tumor’s behavior, making them a valuable tool for studying glioblastoma multiforme (GBM) and the potential treatment strategies. Researchers have three-dimensionally cultured human glioblastoma stem cells (GSCs) by coculturing them with pluripotent stem cell-derived brain organoids. This approach has allowed for a further understanding of the GSC fate behavior and lineage progression within the organoids [71]. Other research teams have also created brain organoids mimicking tumor microenvironments, providing valuable insights into glioblastoma mechanisms and the potential treatment approaches [72]. The effectiveness and safety of tumor-treating fields for glioma treatment have been evaluated using different glioma spheroid cell and brain organoid types, with an aim of exploring the benefits of this treatment strategy [73]. Furthermore, researchers have developed glioblastoma organoids preserving patient-specific tissue and established a pipeline for analyzing cocultured glioblastoma cells [74]. In general, the development and application of human brain organoids in the study of brain cancer, specifically MBs and glioblastomas, have opened new avenues for research. These organoids allow researchers to investigate the genetic mechanisms underlying these cancers, explore cancer stem cell behaviors, and assess potential therapies in a more accurate, representative model system.

## CNS Infectious Diseases

### HIV

Advancements in brain organoid development and composition have provided an opportunity to comprehensively model various neurological complications of HIV. This includes studying the establishment of viral reservoirs and potential curative strategies, as well as the impact of HIV-induced neuroinflammation and neuropathology [75]. Brain organoids, which are 3D cultured models mimicking human brain structure and function, can be used to investigate the effects of antiretroviral toxicity and additional pathologies potentially resulting from substance abuse.

In particular, the use of aged human organoids can help elucidate the combined effects of aging and HIV on the brain, as well as the associated neuropathological mechanisms. Through implementation of previous research on cellular aging and dysfunction, brain organoid models can be used for the assessment of telomere length, formation of toxic protein aggregates (*e.g.*, amyloid β and α-synuclein), and development of dysfunctional autophagy or senescence-activated secretory phenotype [76-78].

Furthermore, iPSC-derived human microglia-containing brain organoids are highly applicable in modeling HIV brain infection [79]. Microglia, the main immune cells in the brain, are also main target cells for HIV infection. Microglial cells, which play a crucial role in drug–HIV-1 interactions, can be studied in brain organoids [80]. Investigating microglial cells within brain organoids can provide a complex cellular environment closely reflecting the actual situation. HIV infection suppresses TLR3 activation–mediated antiviral immunity in microglia and macrophages [81]. Several brain organoid models, such as the MG-Hborg model, support productive viral infection and exhibit an increased inflammatory response by HIV-infected cells, thus mimicking the chronic neuroinflammatory environment observed in individuals with HIV infection [80]. In another study [82], a human brain organoid model was characterized to study HIV infection in the CNS. This model demonstrated that both the brain organoids and the isolated organoid-derived microglia support productive HIV infection via C-C chemokine receptor type 5. This model can be a valuable tool for investigating HIV–CNS interactions, the underlying mechanisms of HIV-associated neurological disorders, and the efficacy of newer therapeutic and curative strategies on the CNS viral reservoir.

In general, the use of human brain organoids demonstrates great promise for gaining a better understanding of HIV infection in the CNS and identifying potential therapeutic targets. Through the study of the viral reservoir composition and activity in the CNS, including the roles of astrocytes and microglia, brain organoids can provide insights into the true nature of latent HIV and its frequency within different cell types.

### COVID-19

COVID-19, caused by SARS-CoV-2, led to the most significant global public health crisis of the 21st century thus far. To gain a comprehensive understanding of symptoms and drug response in patients with COVID-19, studies have used physiologically relevant hESC- or iPSC-derived somatic cells and organoids to mimic human body parts. With these cell models, the authors have replicated the effects of SARS-CoV-2 infection and observed the response of different cell types to the virus [83]. A study assessed the impact of SARS-CoV-2 on the brain using human brain organoids and revealed that the virus primarily infects neurons and has a neurotoxic effect on tau [84]. Another study demonstrated that among all brain cell types, epithelial cells from the choroid plexus—the main blood–cerebrospinal fluid barrier locus—are most susceptible to SARS-CoV-2 infection [85]. These studies have provided valuable insights into the impact of SARS-CoV-2 infection on the brain and facilitated the identification of potential therapeutic targets.

## Exploring Human Brain Development

### Normal Human Brain Development

Brain organoid models enable the simulation of brain development, including neural growth, synapse formation, neural transmission, and cell differentiation. These models aid in understanding the complex mechanisms and patterns of brain development and the interactions between genetic and environmental factors. Studies on immune-driven brain aging have highlighted the potential role of aged monocytes in promoting aging-related markers in brain organoids [86]. Researchers have also developed methods to analyze cell-specific proteins and lipids in brain organoids, highlighting their complexity in mimicking neurodevelopment and aging features [87]. Lineage relationships within these systems can be measured using techniques such as iTracer (reported by He *et al*.), which combines reporter barcodes with CRISPR-Cas9 scarring. These techniques have been used to investigate clonality and lineage dynamics during brain organoid development; they can also be used in any iPSC-derived culture system to elucidate lineage changes during normal or perturbed developmental processes [88]. Furthermore, a combination of tissue expansion and light-sheet fluorescence microscopy enables spatial parameter imaging and quantification during organoid development [33]. These studies have facilitated the exploration of human brain evolution, revealing that brain structure and function are evolutionarily related. They have also been used to explore the mechanisms and driving factors for this evolution and provided strong evidence and theoretical foundation for neuroscience research.

## Congenital Brain Malformations

In 2013, Lancaster *et al*. [10] introduced a method for growing 3D neural tissue from hPSCs. By using this method, the authors created models of congenital brain malformations, caused by disruptions during the embryonic or fetal stages of development [89]. A study incorporated several innovative elements into this approach, building upon existing techniques [90].

## Human Cytomegalovirus

A specific area of interest in studying neurodevelopmental diseases is the role of human cytomegalovirus (HCMV), a leading cause of birth defects in humans. To understand the mechanisms involved further, Ijezie *et al*.[91] used HCMV-infected iPSCs to generate brain organoids in vitro, modeling the first trimester of fetal brain development. The authors found that HCMV infection caused major structural changes in 3D brain organoids. Sun *et al*. [92] demonstrated that developmental impairment due to HCMV can be prevented by neutralizing antibodies that recognize the HCMV pentamer complex.

## Specific Genes

Researchers have also elucidated specific genes involved in brain malformations and their role in neurodevelopment. Dhaliwal *et al*. [93] discovered that in brain organoids, phosphatase and tensin homolog overexpression induces phenotypes similar to microcephaly, a condition characterized by an abnormally small head size. Similarly, Wang *et al*. [94] noted that *NARS1* loss impairs progenitor proliferation in cortical brain organoids, which also leads to microcephaly. Mutations in specific genes can also provide insights into the pathogeneses of several neurodevelopmental disorders. Deng *et al*. [95] constructed mutant brain organoids with a mutation in eukaryotic translation initiation factor 2B to investigate the dynamic process of brain development and vanishing white matter disease. Compared with wild-type brain organoids, mutant brain organoids were noted to exhibit a significantly smaller size and higher apoptosis rate, possibly due to unfolded protein response overactivation.

## Wolfram Syndrome

Brain organoids have also been used to study Wolfram syndrome (WS), a genetic disorder affecting the nervous system. Yuan *et al*. [96] used brain organoids to mimic human brain development; their results indicated that WFS1-deficient organoids replicated the neuronal loss observed in patients with WS. The authors also noted impaired synapse formation and function along with reduced astrocytes. These insights into the effects of WFS1 deficiency on synapse formation and function may guide future WS treatment strategies. Furthermore, Takla *et al*.[97] used a self-organizing single rosette spheroid (SOSRS) brain organoid system to study the genetic basis of teratogenesis in neural tube defects (NTDs) and the underlying mechanisms. By developing a high-throughput image analysis pipeline, the authors assessed NTD-like phenotypes in the SOSRS structures, effectively reflecting human neurodevelopment.

In general, the use of brain organoids provides a potential platform for studying the pathological mechanisms underlying neurodevelopmental diseases. This progress in research techniques allow for a better understanding of brain development and offers opportunities for developing treatments for various disorders.

By utilizing these organoids, we can delve into the underlying mechanisms and pathological processes of neurological disorders.

## Conclusions and Perspectives

Recent improvements in brain organoid technologies have led to groundbreaking discoveries and achievements. While brain organoids show significant potential, improving scalability and functional maturity is crucial for their broader application. By addressing current limitations and creating improved organoids, researchers may enhance the current understanding of brain development and disorders. These improvements can closely resemble the human brain at a higher level. Although these organoids have practical applications, avoiding overhyping the technology and raising false hopes are essential [98]. This progress may help researchers uncover mechanisms underlying specific neurological conditions and disorders, particularly those involving early developmental processes.

By creating organoids accurately replicating the structure, cellular composition, electrical signaling, and functional connectivity of the human brain, researchers will have a powerful tool to study brain development and investigate various brain disorders, such as neurodegenerative disorders, nervous system trauma, brain cancer, and CNS infectious diseases. Moreover, some researchers (*e.g.*, Smirnova *et al*. [99]) believe that by integrating cutting-edge artificial intelligence with brain organoid research, organoid intelligence can be established as a form of synthetic biological intelligence. Moreover, Kagan *et al*. [100] demonstrated that when embodied in a simulated game world, in vitro neurons can exhibit learning capabilities and sentience. Nonetheless, these findings have led to a debate on the potential consciousness of these organoids [101-104].

Further research on understanding whether these brain organoids have the aforementioned abilities and determining whether these abilities imply the emergence of consciousness is warranted. Ethical considerations related to the use of organoid consciousness, in spite of that, remain unclear. In this rapidly evolving field, understanding the possibilities and limitations of organoid consciousness is crucial for guiding further scientific experimentation and shaping the related ethical guidelines. In general, these advancements provide newer avenues for the exploration and understanding of the complexities of the human brain.

## Supplemental Materials

Supplementary data for this paper are available on-line only at http://jmb.or.kr.



## Figures and Tables

**Table 1:** Advances and limitations in brain organoid research.

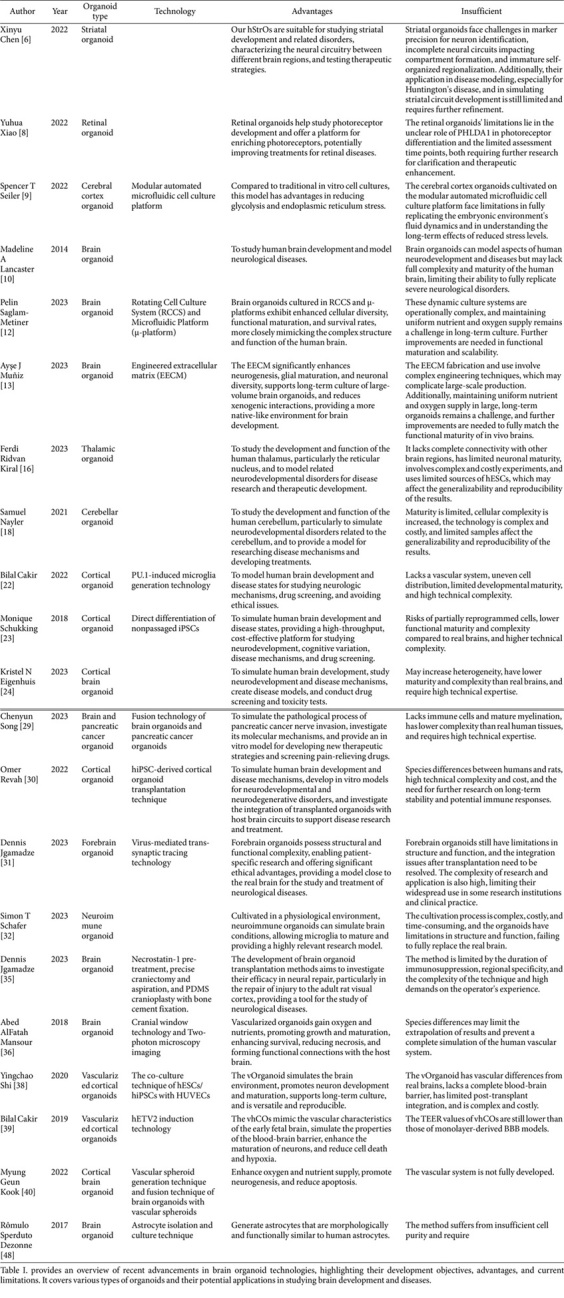

**Table 2:** Applications and limitations of brain organoids in disease modeling.