# Supplementary material for: Human Brain Organoids: Development and Applications
Source: J Microbiol Biotechnol. 2025 May 28;35:e2411040. doi: 10.4014/jmb.2411.11040 (PMC12149405; doi:10.4014/jmb.2411.11040)
Supplement: Supplementary file 1 [file jmb-35-e2411040-supple.pdf]

Table S1. Search strategy

No	Search items
#1	"Brain"[Mesh]
#2	(Brain[Title/Abstract]) OR (Encephalon[Title/Abstract])
#3	#1 OR #2
#4	"Organoids"[Mesh]
#5	(Organoids[Title/Abstract]) OR (Organoid[Title/Abstract])
#6	#4 OR #5
#7	#3 AND #6
#8	"Cerebrum"[Mesh]
#9	(((Cerebrum[Title/Abstract]) OR (Cerebra[Title/Abstract])) OR (Cerebral Hemispheres[Title/Abstract])) OR (Cerebral Hemisphere[Title/Abstract])
#10	#8 OR #9
#11	#10 AND #6
#12	"Disease"[Mesh]
#13	(Disease[Title/Abstract]) OR (Diseases[Title/Abstract])
#14	#12 OR #13
#15	Model[Title/Abstract]
#16	#14 AND #15
#17	#7 AND #16
#18	#10 AND #16
#19	#17 OR #18
